# Sequential embryo transfer versus double cleavage-stage embryo or double blastocyst transfer in patients with recurrent implantation failure with frozen-thawed embryo transfer cycles: a cohort study

**DOI:** 10.3389/fendo.2023.1238251

**Published:** 2023-09-08

**Authors:** Jiangman Gao, Yifeng Yuan, Jia Li, Tian Tian, Ying Lian, Ping Liu, Rong Li, Jie Qiao, Xiaoyu Long, Haiyan Wang

**Affiliations:** ^1^ State Key Laboratory of Female Fertility Promotion, Center for Reproductive Medicine, Department of Obstetrics and Gynecology, Peking University Third Hospital, Beijing, China; ^2^ National Clinical Research Center for Obstetrics and Gynecology Department of Obstetrics and Gynecology, Peking University Third Hospital, Beijing, China; ^3^ Key Laboratory of Assisted Reproduction (Peking University), Ministry of Education, Department of Obstetrics and Gynecology, Peking University Third Hospital, Beijing, China; ^4^ Beijing Key Laboratory of Reproductive Endocrinology and Assisted Reproductive Technology, Department of Obstetrics and Gynecology, Peking University Third Hospital, Beijing, China

**Keywords:** repeated implantation failure, frozen-thawed embryo transfer, sequential embryo transfer, cleavage-stage embryo transfer, blastocyst transfer

## Abstract

**Background:**

Recurrent implantation failure (RIF) is more common among patients receiving assisted reproductive treatment. Many efforts have been made to increase the incidence of clinical pregnancy among patients with RIF. The effect of the sequential transfer procedure, a two-step interval transfer of a cleavage-stage embryo followed by a blastocyst in one transfer cycle, on the clinical outcomes of RIF patients remains controversial.

**Methods:**

In total, 1774 frozen-thawed embryo transfer (FET) cycles in RIF patients were included. Of these cycles, 302 were sequential embryo transfer (ET) cycles, 979 were double day 3 cleavage-stage ET cycles, and 493 were double blastocyst ET cycles. The primary outcomes were the rates of implantation, clinical pregnancy and multiple pregnancy, and the secondary outcomes were the rates of hCG positive, early miscarriage and ectopic pregnancy.

**Results:**

The implantation, hCG positive, and clinical pregnancy rates in the sequential ET group (32.1%, 58.9%, 50.7%) were significantly higher than those in the day 3 cleavage-stage ET group (24.9%, 46.5%, 40.4%) and were similar to those in the blastocyst transfer group (30.1%, 56.4%, 47.1%). The early miscarriage rate in the blastocyst transfer group was significantly higher than that in the cleavage-stage ET group (17.2% vs. 8.1%, *P <*0.05), while the ectopic pregnancy rate in the blastocyst transfer group was significantly lower than that in the cleavage-stage ET group (0.4% vs. 3.0%, *P <*0.05). The multiple pregnancy rate in the sequential ET group was significantly lower than that in the cleavage-stage ET group (17.0% vs. 25.5%, *P <*0.05) and the blastocyst transfer group (17.0% vs. 27.6%, *P <*0.05). When cycles of blastocyst culture failure were excluded, the clinical pregnancy rate was significantly higher (55.7% vs. 47.1%, *P <*0.05), and the early miscarriage rate and multiple pregnancy rate were significantly lower (8.5% vs. 17.2%, 17.7% vs. 27.6%; *P <*0.05, respectively) in the sequential ET group than in the double blastocyst ET group.

**Conclusions:**

Sequential embryo transfer in FET cycles could improve the clinical outcomes of patients with RIF.

## Introduction

Since 1978, many infertile couples have benefited from assisted reproductive technology (ART). As of 2019, more than 8 million children had been born after ART worldwide. Over 2.5 million *in vitro* fertilization (IVF) cycles are performed every year, resulting in over 500,000 deliveries annually (1). However, the best embryo implantation rate ranges from 25-40% (2, 3). Improving the success rate is a challenging problem associated with ART treatment programs.

Repeated implantation failure (RIF) refers to a situation when the transferred embryos fail to implant after at least three IVF-embryo transfer (IVF-ET) cycles with 1-2 high-quality embryos in each cycle (4, 5). The prevalence of RIF is 8-15% (6–8), which poses great difficulties and challenges to clinicians and embryologists. Recurrent failures of IVF-ET also bring psychological, physical, and financial distress to patients.

High-quality embryos, a receptive endometrium, and good synchrony between the embryo and endometrium are necessary conditions for successful implantation (9). The implantation process involves three phases: apposition, adhesion, and invasion. During these stages, the cross-talk between the endometrium and embryo is significant, and suboptimal endometrial receptivity is the most critical cause of RIF (10).

In recent years, scientists have proposed a sequential transfer procedure, a two-step interval transfer of a cleavage-stage embryo followed by a blastocyst in one transfer cycle to help RIF patients increase the chance of pregnancy. Several studies have suggested that sequential ET significantly improves the clinical outcomes of IVF-ET (11–13). However, the effect of sequential ET is still controversial, and its effectiveness and potential biological mechanisms have not been proven.

To further confirm the effect of sequential ET, this study analyzed the data of sequential ET at our reproductive center to evaluate the effect of sequential ET on the clinical outcomes of patients with a history of RIF in frozen-thawed embryo transfer (FET) cycles. The primary outcome measures were implantation rate, clinical pregnancy rate and multiple pregnancy rate. The secondary outcome measures were hCG positive rate, early miscarriage rate and ectopic pregnancy rate.

## Patients and methods

This retrospective observational study was performed at the Reproductive Center of Peking University Third Hospital from January 2020 to June 2022. Patients who had not conceived after three or more ET cycles and undergone sequential ET (one day 3 cleavage-stage embryo followed by a day 5/6 blastocyst in one FET cycle) were included (302 cycles). Two groups based on the ET strategy were adopted as the control groups: the double cleavage-stage embryo (day 3) transfer group (979 cycles) and the double blastocyst (day 5/6) transfer group (493 cycles). Among these participants, those employing PGT for chromosomal structural rearrangements (PGT-SR) or monogenic/single gene defects (PGT-M), those using egg donor cycles, those with a thin endometrium (thickness less than 6 mm) and those with autoimmune diseases were excluded.

### Frozen-thawed embryo transfer procedure

All the included cycles were FET cycles. Endometrial preparation for FET was performed as previously described (14), and the preparation method was the artificial (hormone replacement) cycle, natural cycle, or stimulation cycle. On the transfer day, embryo grading was performed. Cleavage-stage embryos were evaluated according to the criteria of the Istanbul Embryo Evaluation Symposium (15), and blastocysts were evaluated using the Gardner grading system (16). The embryos were transferred using the Cook Sydney IVF catheter (k-jets-7019-SIVF). In the sequential ET group, one of the frozen-thawed embryos was transferred on day 3, whereas the rest were cultured; then, one blastocyst was transferred on day 5 or day 6. In the cleavage-stage ET group, double embryos were transferred on day 3, while in the blastocyst ET group, double blastocysts were transferred on day 5.

### Outcome measures

Serum β-hCG levels were measured 14-21 days after ET, with β-hCG levels ≥25 IU/L being defined as biochemical pregnancy, also named hCG positive. Clinical pregnancy is defined as the presence of an intrauterine gestational sac on ultrasonography. The implantation rate was defined as the number of gestational sacs divided by the total number of embryos or blastocysts transferred. Early miscarriage was defined as loss of the clinical pregnancy within 12 weeks of gestation. Ectopic pregnancy was defined as an extrauterine pregnancy. Multiple pregnancy was defined as the presence of two or more gestational sacs on ultrasound, and the rate was calculated as the number of multiple pregnancy cycles divided by the number of clinical pregnancy cycles.

### Statistical analysis

Data analysis was performed using SPSS statistics software version 23 (IBM). The continuous variables are presented as the means ± standard deviations (SDs). One-way ANOVA was used for continuous variables that had a normal distribution, while the Kruskal−Wallis test was performed for nonnormally distributed continuous data. Categorical variables are presented as counts and percentages. The chi-square test was applied to test categorical variables. In the multivariate logistic regression analysis, FET groups, parental age, infertility duration, cycles of implantation failure, insemination methods, endometrial preparation methods and endometrial thickness were included, and adjusted odds ratios (ORs) and 95% confidence intervals (95% CIs) were reported. A *P* value of < 0.05 was considered statistically significant for all tests.

## Results

### Characteristics of sequential embryo transfer cycles

**Figure 1:**
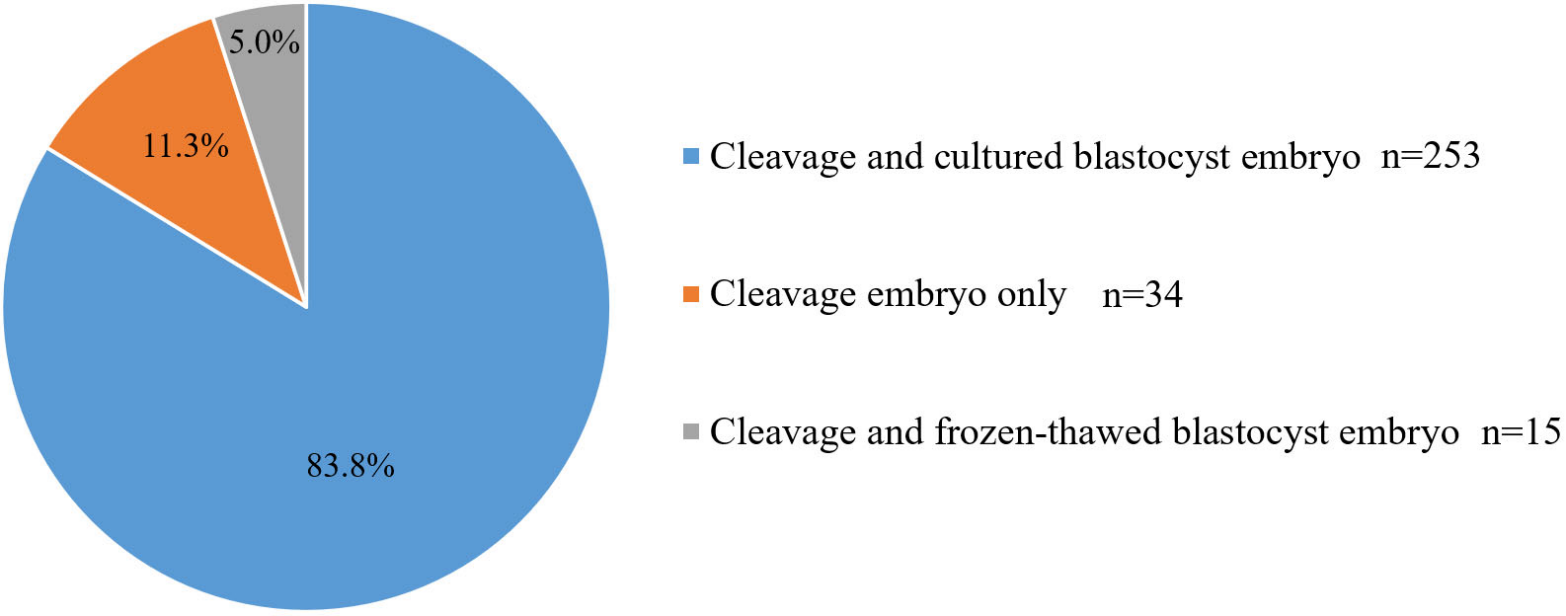
The cycles of different transferred embryos in the sequential ET.

A total of 302 sequential ET cycles were included in this study. Of these, there were 5 cycles with no embryo to culture after cleavage-stage ET, resulting in 2 cycles of cleavage-stage ET only and 3 cycles of cleavage-stage ET followed by frozen-thawed blastocyst transfer. There were 44 cycles in which the remaining frozen-thawed cleavage-stage embryos were not cultured into blastocysts after cleavage-stage ET (including 32 cycles of cleavage-stage ET only and 12 cycles of cleavage-stage ET followed by frozen-thawed blastocyst transfer). Overall, 253 cycles were completed successfully with day 3 cleavage-stage ET followed by cultured blastocyst transfer (**Table 1**). In total, day 3 cleavage-stage embryo and cultured blastocyst transfer cycles, day 3 cleavage-stage embryo and frozen-thawed blastocyst transfer cycles, and day 3 cleavage-stage ET only cycles accounted for 83.8%, 5.0%, and 11.3% of total sequential ET cycles, respectively (**Figure 1**).

**Table 1:** Cycle characteristics of sequential embryo transfer of FET.

Sequential embryo transfer	Cycles (n)	Clinical pregnancy n (%)
Total cycles	**302**	**153(50.7%)**
No embryo to culture after a cleavage-stage embryo transfer	**5**	**1(20%)**
A cleavage-stage embryo transfer only	2	0 (0)
A cleavage-stage embryo transfer followed by a frozen-thawed blastocyst transfer	3	1(33.3%)
Frozen-thawed cleavage-stage embryos did not form blastocysts after a cleavage-stage embryo transfer	**44**	**11(25%)**
A cleavage-stage embryo transfer only	32	8(25%)
A cleavage-stage embryo transfer followed by a frozen-thawed blastocyst transfer	12	3(25%)
A cleavage-stage embryo transfer followed by a cultured blastocyst transfer	**253**	**141(55.7%)**

The bold values in **Table 1** indicate the primary classification; non-bold values are the further subgroups.

In summary, of the 302 sequential ET cycles performed, cleavage-stage embryos were transferred in only 34 (11.3%) cycles due to the lack of embryos for culture after ET (2 cycles) or failure of the remaining embryos to form blastocysts (32 cycles). There were fifteen (5.0%) cycles of cleavage-stage ET followed by frozen-thawed blastocyst transfer. In addition, 253 (83.8%) cycles were completed successfully with day 3 cleavage-stage ET followed by cultured blastocyst transfer.

### Demographic characteristics of the three groups

We compared the baseline data, including parental age, female BMI, infertility duration, primary infertility ratio, previous failed cycles, insemination method, endometrial preparation protocols, and endometrial thickness on FET among the three groups. The infertility duration was significantly shorter (4.91 ± 3.29 years vs. 5.52 ± 3.27 years & 5.73 ± 3.46 years), and the number of cycles of previous failure were significantly lower (3.14 ± 1.69 vs. 4.83 ± 2.36 & 4.07 ± 2.00) in the cleavage-stage ET group than in the sequential ET and blastocyst transfer groups. The proportion of artificial cycles was significantly higher (65.9% vs. 53.7% & 56.2%) in the sequential ET group than in the cleavage-stage ET and blastocyst transfer groups. There were no significant differences in parental age, female BMI, primary infertility ratio, insemination method, and endometrial thickness on FET among the three groups (**Table 2**).

**Table 2:** Demographic and cycle characteristics among the three groups according to embryo transfer of FET.

Variable	Sequential embryo transfer (n = 302)	Cleavage-stage embryo transfer (n = 979)	Blastocyst embryo transfer (n = 493)	*P* value
Female age (years)	34.05 ± 4.51	33.63 ± 4.27	33.67 ± 4.01	0.302
Male age (years)	35.13 ± 6.82	34.94 ± 5.34	34.88 ± 5.13	0.291
Body mass index	22.39 ± 3.48	22.37 ± 3.45	22.36 ± 3.23	0.993
Infertility duration (years)	5.52 ± 3.27	4.91 ± 3.29	5.73 ± 3.46	<0.001*
Primary infertility ratio (n, %)	201 (66.6%)	650 (66.4%)	320 (64.9%)	0.831
Previous failed cycles (n)	4.83 ± 2.36	3.14 ± 1.69	4.07 ± 2.00	<0.001*
Insemination method (n, %)				0.246
IVF	202 (66.9%)	603 (61.6%)	312 (63.3%)	
ICSI	100 (33.1%)	376 (38.4%)	181 (36.7%)	
Endometrial preparation (n, %)				0.006*
Artificial cycle	199(65.9%)	526 (53.7%)	277(56.2%)	
Natural cycle	88 (29.1%)	378 (38.6%)	185 (37.5%)	
Stimulation cycle	15 (5.0%)	75 (7.7%)	31 (6.3%)	
Endometrial thickness on FET (mm)	9.99 ± 1.73	9.98 ± 1.79	10.07 ± 1.67	0.757

IVF, in vitro fertilization; ICSI, intracytoplasmic sperm injection; FET, frozen-thawed embryo transfer; *P<0.05.

The bold values in **Tables 2**, **3** indicate significant statistical differences among groups (P <0.05).

### FET outcomes

**Figure 2:**
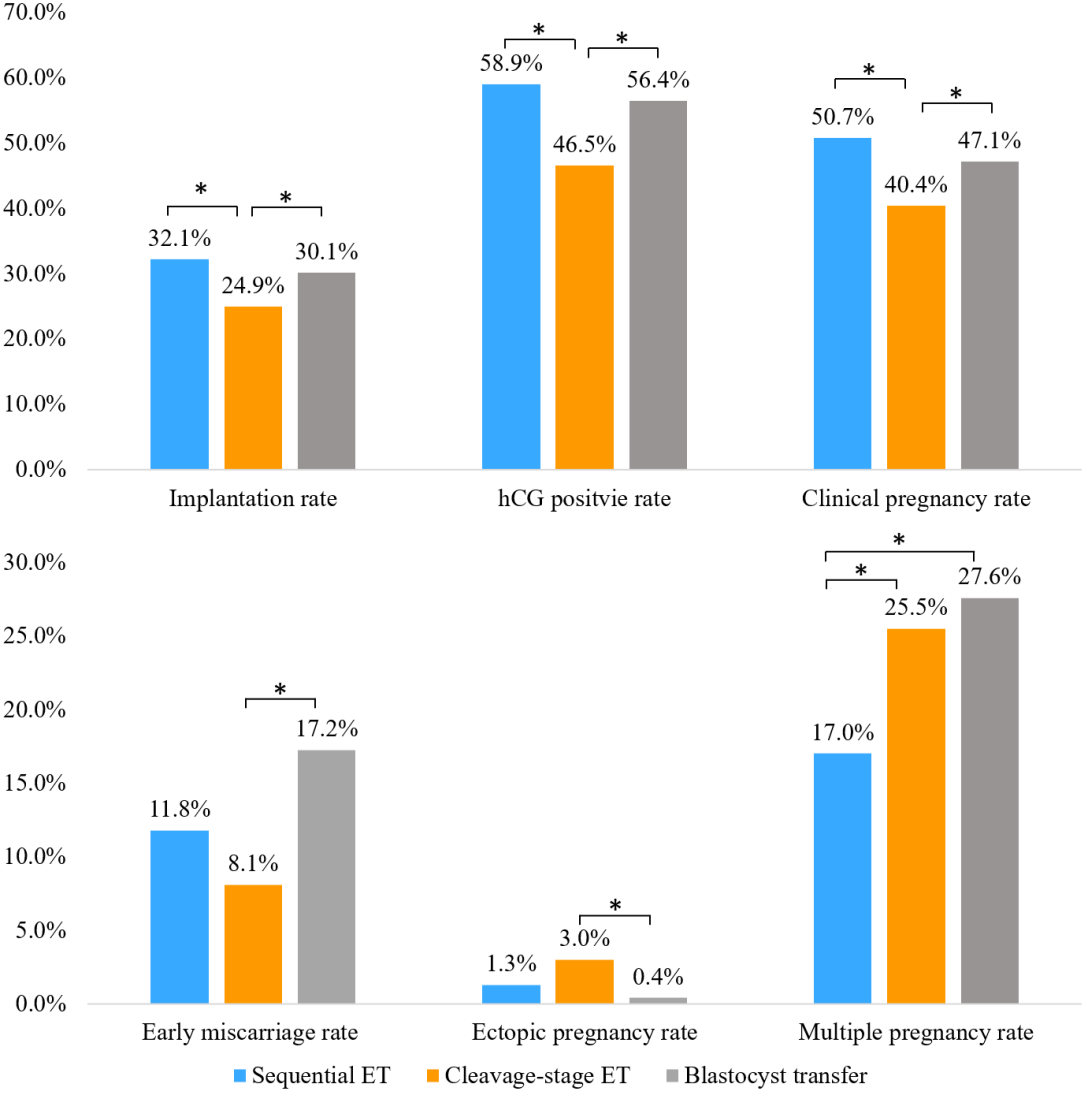
Clinical outcomes of patients in each group. *P<0.05.

Compared to those in the cleavage-stage ET group, the implantation, hCG positive, and clinical pregnancy rates were significantly higher in the sequential ET group and the blastocyst transfer group. There was no significant difference in the implantation rate, hCG positive rate, or clinical pregnancy rate between the sequential ET group and the blastocyst transfer group. The early miscarriage rate in the blastocyst transfer group was significantly higher than that in the cleavage-stage ET group, while the ectopic pregnancy rate in the blastocyst transfer group was significantly lower than that in the cleavage-stage ET group. The early miscarriage rate and ectopic pregnancy rate in the sequential ET group were not significantly different from the rates in the other two groups. The multiple pregnancy rate in the sequential ET group was significantly lower than that in the cleavage-stage ET group and the blastocyst transfer group (**Figure 2**).

**Figure 3:**
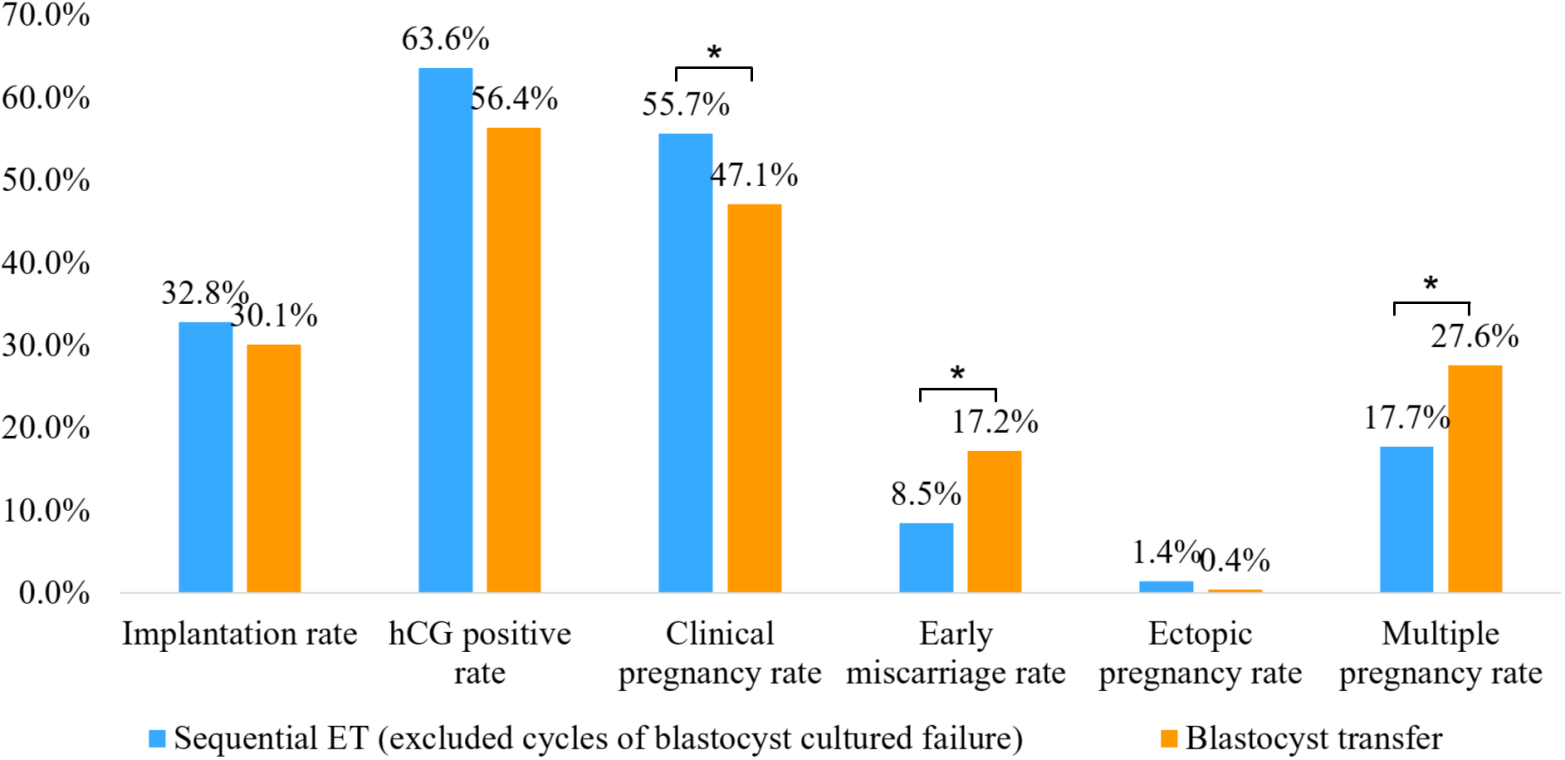
Comparison of clinical outcomes between successfully completed sequential embryo transfer group (day 3 cleavage-stage embryo transfer followed by a cultured blastocyst transfer) and double blastocyst transfer group. *P<0.05.

We then compared the clinical outcomes of cycles in which sequential ET was completed successfully (a day 3 cleavage-stage ET followed by a cultured blastocyst transfer) with those of cycles in which blastocyst transfer had been completed successfully. The clinical pregnancy rate in the successfully completed sequential ET group was significantly higher than that in the blastocyst transfer group, while the early miscarriage rate and multiple pregnancy rate in the successfully completed sequential ET group were significantly lower than those in the blastocyst transfer group. There was no significant difference in the implantation rate, hCG positive rate, or ectopic pregnancy rate between the two groups (**Figure 3**).

Multiple logistic regression analysis with adjustments for possible confounders for clinical pregnancy, early miscarriage, and multiple pregnancy was used to evaluate the effectiveness of sequential ET (**Table 3**). Adjustments were made for confounding variables including FET group, parental age, infertility duration, cycles of implantation failure, insemination methods, endometrial preparation methods, and endometrial thickness. Compared to sequential ET and double blastocyst transfer, double cleavage-stage ET had a significantly lower clinical pregnancy rate (OR 0.610, 95% CI 0.432-0.861, *P*=0.005; OR 0.697, 95% CI 0.531-0.914, *P*=0.009, respectively). Endometrial thickness was associated with clinical pregnancy (OR 1.087, 95% CI 1.016-1.164, *P*=0.016). Double cleavage-stage ET had a lower early miscarriage rate than double blastocyst transfer (OR 0.439, 95% CI 0.240-0.800, *P*=0.007), female age had a significantly negative effect on early miscarriage (OR 1.162, 95% CI 1.040-1.299, *P*=0.008), and double blastocyst transfer had a significantly higher multiple pregnancy rate than sequential ET (OR 1.860, 95% CI 1.012-3.418, *P*=0.046).

**Table 3:** Multivariate logistic regression analysis of clinical outcomes after adjustment.

	Adjusted OR	95% CI	*P* value
Clinical pregnancy			
FET group (cleavage vs. sequential)	0.610	0.432-0.861	0.005*
FET group (blastocyst vs. sequential)	0.875	0.612-1.251	0.465
FET group (cleavage vs. blastocyst)	0.697	0.531-0.914	0.009*
Female age	0.965	0.924-1.007	0.104
Male age	0.983	0.951-1.016	0.317
Infertility duration	0.964	0.927-1.003	0.069
Previous failed cycles	1.005	0.942-1.072	0.880
Insemination method (ICSI vs. IVF)	0.979	0.766-1.252	0.866
Endometrial preparation (natural vs. artificial)	1.169	0.911-1.500	0.222
Endometrial preparation (stimulation vs. artificial)	0.864	0.528-1.415	0.562
Endometrial preparation (natural vs. stimulation)	1.352	0.815-2.243	0.242
Endometrial thickness on FET	1.087	1.016-1.164	0.016*
Early miscarriage			
Group of FET (cleavage vs. sequential)	0.790	0.349-1.788	0.572
Group of FET (blastocyst vs. sequential)	1.802	0.824-3.942	0.140
Group of FET (cleavage vs. blastocyst)	0.439	0.240-0.800	0.007*
Female age	1.162	1.040-1.299	0.008*
Male age	0.929	0.850-1.017	0.110
Infertility duration	0.992	0.904-1.089	0.868
Previous failed cycles	0.862	0.731-1.018	0.079
Insemination method (ICSI vs. IVF)	1.197	0.694-2.063	0.518
Endometrial preparation (natural vs. artificial)	0.595	0.329-1.076	0.086
Endometrial preparation (stimulation vs. artificial)	1.596	0.599-4.322	0.358
Endometrial preparation (natural vs. stimulation)	0.373	0.129-1.073	0.067
Endometrial thickness on FET	0.978	0.842-1.137	0.776
Multiple pregnancy			
Group of FET (cleavage vs. sequential)	1.315	0.714-2.421	0.380
Group of FET (blastocyst vs. sequential)	1.860	1.012-3.418	0.046*
Group of FET (cleavage vs. blastocyst)	0.707	0.448-1.115	0.135
Female age	0.950	0.872-1.034	0.234
Male age	0.994	0.929-1.065	0.871
Infertility duration	1.008	0.935-1.086	0.837
Previous failed cycles	1.025	0.916-1.147	0.665
Insemination method (ICSI vs. IVF)	0.786	0.513-1.205	0.270
Endometrial preparation (natural vs. artificial)	0.950	0.623-1.449	0.811
Endometrial preparation (stimulation vs. artificial)	0.319	0.093-1.089	0.068
Endometrial preparation (natural vs. stimulation)	2.980	0.860-10.326	0.085
Endometrial thickness on FET	0.951	0.844-1.075	0.434

IVF, in vitro fertilization; ICSI, intracytoplasmic sperm injection; FET, frozen-thawed embryo transfer; OR, odds ratio; CI, confidence interval; *P<0.05.

The bold values in **Tables 2**, **3** indicate significant statistical differences among groups (P <0.05).

## Discussion

Sequential ET was first performed by Abramovici et al. in 1988 since embryo freezing was not an option in their IVF-ET program at that time (17). Since then, some clinicians have tried to use a sequential ET approach to help patients with RIF increase their chances of pregnancy. Some studies showed that sequential ET did not improve clinical outcomes for patients with RIF (18–20), while other studies suggested that it was more effective toward increasing the clinical pregnancy and live birth rates than day 3 or day 5 ET in these patients (11–13). The study by Ji et al. included patients with a history of RIF undergoing FET cycles (18), and studies by Tehraninejad et al. (20) and Kyono et al. (19) included patients undergoing IVF fresh cycles and showed that sequential embryo transfer failed to increase the chance of pregnancy. A retrospective cohort study by Stamenov et al. (12) and a prospective and randomized trial by Torky et al. (13) showed that the implantation and clinical pregnancy rates were significantly higher in patients who underwent sequential embryo transfer than in those who underwent cleavage transfer on day 3 or blastocyst transfer on day 5 in IVF fresh cycles. Arefi et al. (11) conducted a prospective study to evaluate the improvement of pregnancy rate in sequential FET on day 3/day 5 in individuals who suffered from RIF and suggested that sequential transfer was more effective than regular day 5. A systematic review by Zhang et al. demonstrated that the clinical pregnancy rate and live birth rate were higher in the sequential ET group than in the cleavage-stage ET group for women who experienced RIF, and there were no significant differences between sequential ET and blastocyst ET (21). Even in patients with poor ovarian response, a report showed that sequential transfer had a higher live birth rate than day 3 ET and had a similar live birth rate to blastocyst transfer in the FET cycle (14). However, the sample size in each study was less than 150 subjects. In the present retrospective study, we included 302 cycles as the observation group. We also included 979 cycles of double day 3 ET and 493 cycles of double blastocyst transfer as the control groups. All of the patients had a history of three consecutive implantation failures and underwent FET cycles. We found that sequential ET had higher rates of implantation and pregnancy than conventional cleavage-stage ET. In addition, the rates of implantation and pregnancy in the sequential ET group were similar to those in the double blastocyst transfer group without increasing the risk of early miscarriage or ectopic pregnancy. This finding suggests that sequential ET may be effective in improving pregnancy outcomes in patients with RIF.

There are several advantages and potential mechanisms in sequential ET. First, successful embryo implantation requires synchronous interactions between endometrial receptivity and embryos with high developmental potential. During the implantation process, many molecular mediators, including cytokines, lipids, adhesion molecules, growth factors, and others, support the establishment of pregnancy (9). Endometrial injury by biopsy catheters during the luteal phase of the menstrual cycle has been shown to improve implantation and pregnancy rates in subsequent treatment cycles (22). During the window of implantation, endometrial preparation is guided not only by maternal factors but also by molecules secreted by the embryo, such as chorionic gonadotropin and interleukin-1β (IL-1 β) (23). Therefore, after the first ET procedure of sequential ET, mechanical microstimulation caused by catheter insertion and cytokines produced by the embryo and endometrium may not only be a benefit for the implantation of the first transferred embryo but could also promote better implantation conditions and increase the implantation probability following blastocyst transfer (24). Second, the window of implantation is transient in humans, and implantation beyond this window results in pregnancy failure (25). Mechanical stimulation of the endometrium may slightly alter the implantation window for personalized ET, which has a beneficial effect on the receptive endometrium (26). In addition, sequential ET can probably extend the availability time for transferred embryos to access the implantation window. Moreover, compared with double cleavage-stage day 3 ET, cleavage-stage embryos cultured *in vitro* from 3 days to 5-6 days could be screened to identify embryos with higher implantation potential, resulting in a higher pregnancy rate (27).

Compared with double blastocyst transfer, sequential ET can decrease the risk of ET cycle cancelation since prolonged culture may result in a lack of available blastocysts for transfer. In this study, the clinical pregnancy rate was approximately 25%, even when only a single day 3 embryo was transferred during sequential ET cycles. For patients with many embryos that have good developmental potential, sequential ET is likely to help them improve clinical outcomes. Our study showed that the clinical pregnancy rate was significantly higher and that the early miscarriage rate was significantly lower in the successfully completed sequential ET treatment (a day 3 cleavage-stage ET followed by transfer of a cultured blastocyst) than in the double blastocyst ET treatment. This further suggests that the first day 3 cleavage-stage ET procedure in sequential ET could probably improve the clinical outcomes of ART treatment.

The prevalence of multiple pregnancy is higher with ART than with natural pregnancy (28). This is related to the number of ETs, and there is a consequent impact on maternal and newborn outcomes (29, 30). In the last two decades, controlling the number of embryos transferred (single ET per cycle) has been advocated to reduce the risks of multiple gestations (28, 31). However, compared to the successive failure of IVF-ET, increasing the probability of pregnancy is more beneficial to patients economically and psychologically, even though it sometimes increases the risk of multiple pregnancy. In the case of multiple pregnancy, multifetal pregnancy reduction is also an option for patients to reduce the risk of maternal and fetal complications (32). Therefore, transferring two embryos is a strategy used for patients with RIF. In contrast to previous studies, this study showed that the multiple pregnancy rate in the sequential ET group was significantly lower than that in the double cleavage-stage ET group and the double blastocyst group. This seems to be another benefit of sequential ET. However, further studies are needed to investigate and define multiple pregnancy occurrence in sequential ET.

The larger population and two types of ET (double cleavage-stage embryos and double blastocysts) as controls are the strengths of our study. However, it also had some limitations, including its retrospective nature and lack of data related to live birth. Prospective studies are needed to identify this scientific issue to meet the clinical demand.

In conclusion, this study investigated the value of sequential ET in patients with RIF in FET cycles. Sequential ET was associated with a higher implantation rate, hCG positive rate, and clinical pregnancy rate than double cleavage-stage ET and comparable to those of the double blastocyst-stage ET group without increasing the risk of early miscarriage or ectopic pregnancy. Sequential ET had a lower multiple pregnancy rate than double cleavage-stage ET and double blastocyst-stage ET. In addition, the clinical pregnancy rate was significantly higher, and the early miscarriage rate was significantly lower in the sequential ET than in the double blastocyst-stage ET when cycles of blastocyst culture failure in the sequential ET group were excluded. These findings suggested that sequential ET is an effective and beneficial option for patients with RIF.

## Data availability statement

The original contributions presented in the study are included in the article. Further inquiries can be directed to the corresponding authors.

## Ethics statement

The studies involving humans were approved by Peking University Third Hospital Medical Science Research Ethics Committee (M2022128). The studies were conducted in accordance with the local legislation and institutional requirements. Written informed consent for participation was not required from the participants or the participants’ legal guardians/next of kin in accordance with the national legislation and institutional requirements.

## Author contributions

HW and XL conceived the idea. JG and JL reviewed the literature and designed the study. YY and TT collected the data and conducted the analysis. JG designed the figures and tables and wrote the manuscript. YL, PL, RL, JQ, HW and XL coordinated the study and revised the manuscript. All authors contributed to the article and approved the submitted version.
